# Bioresorbable Scaffolds for Coronary Revascularization: From Concept to Clinical Maturity

**DOI:** 10.3390/jcdd13010002

**Published:** 2025-12-19

**Authors:** Angeliki Bourazana, Alexandros Briasoulis, Christos Kourek, Toshiki Kuno, Ioannis Leventis, Chris Pantsios, Vasiliki Androutsopoulou, Kyriakos Spiliopoulos, Grigorios Giamouzis, John Skoularigis, Andrew Xanthopoulos

**Affiliations:** 1 Department of Cardiology, General Hospital of Larissa, 41221 Larissa, Greece; angi3bou@gmail.com; 2 Department of Cardiovascular Medicine, Section of Heart Failure and Transplantation, University of Iowa, Iowa City, IA 52242, USA; 3 Department of Cardiology, 417 Army Share Fund Hospital of Athens (NIMTS), 11521 Athens, Greece; 4 Division of Cardiology, Massachusetts General Hospital, Harvard Medical School, Boston, MA 02114, USA; 5 Department of Cardiology, University Hospital of Larissa, Faculty of Medicine, School of Health Sciences, University of Thessaly, 41100 Larissa, Greecegrgiamouzis@gmail.com (G.G.);; 6 Department of Cardiac Surgery, University Hospital of Larissa, University of Thessaly, 41110 Larissa, Greece; androutsopoulouvasiliki@hotmail.com (V.A.);

**Keywords:** stents, drug-eluting, bioresorbable scaffolds, revascularization

## Abstract

Over the past decades, coronary revascularization has evolved dramatically with the introduction of bioresorbable scaffolds (BRSs), designed to provide temporary vessel support, elute antiproliferative drugs, and then fully resorb, ideally restoring natural vasomotion and eliminating long-term foreign-body reactions. Early enthusiasm for first-generation polymeric devices, such as the Absorb bioresorbable vascular scaffold, was tempered by increased rates of scaffold thrombosis and late adverse events, largely attributed to thick struts, suboptimal implantation techniques, and unpredictable degradation kinetics. Subsequent developments in polymeric (e.g., MeRes-100, NeoVas) and metallic magnesium-based scaffolds (e.g., Magmaris) have focused on thinner struts, improved radial strength, and refined resorption profiles. Clinical trials and meta-analyses, including ABSORB, AIDA, BIOSOLVE, and BIOSTEMI, reveal that optimized procedural strategies, especially the “PSP” approach (Prepare–Size–Post-dilate) and routine intravascular imaging, substantially reduce thrombosis and restenosis rates, aligning outcomes closer to those of contemporary drug-eluting stents (DESs). Nonetheless, challenges persist regarding inflammatory responses to degradation by-products, mechanical fragility in complex lesions, and patient selection. Ongoing innovations include hybrid polymer–metal designs, stimuli-responsive drug coatings, and AI-assisted imaging for precision implantation. While early-generation BRSs demonstrated both promise and pitfalls, next-generation platforms show steady progress toward achieving the dual goals of transient scaffolding and long-term vessel restoration. The current trajectory suggests that bioresorbable technology, supported by optimized technique and material science, may soon fulfill its original vision; offering safe, effective, and fully resorbable alternatives to permanent metallic stents in coronary artery disease. This review provides an updated synthesis of the design principles, clinical outcomes, and procedural considerations of drug-eluting bioresorbable scaffolds (BRSs). It integrates recent meta-analytic evidence and emerging insights on device mechanics, including the influence of strut thickness on radial strength and the potential role of non-invasive imaging in pre-implantation planning. Special focus is given to magnesium-based scaffolds and future directions in patient selection and implantation strategy.

## 1. Introduction

Over the past four decades, the treatment of coronary artery disease (CAD) has undergone significant transformation through advancements in percutaneous coronary intervention (PCI). Bare-metal stents (BMSs) emerged as an early solution to mitigate the limitations of balloon angioplasty, such as acute vessel recoil and early restenosis. Landmark trials in the 1990s demonstrated the superiority of BMSs over balloon angioplasty in reducing restenosis rates [1]. However, BMSs were soon associated with in-stent restenosis due to neointimal proliferation. To address these limitations, drug-eluting stents (DESs) were developed, combining mechanical scaffolding with antiproliferative drug release. First-generation DESs markedly reduced target lesion revascularization (TLR) and in-stent restenosis (ISR), representing a major advancement over BMSs [2]. Subsequent generations introduced more biocompatible polymers and thinner struts, leading to further safety and efficacy improvements [3]. Nevertheless, long-term concerns persisted regarding delayed endothelialization, chronic inflammation, stent thrombosis, and permanent caging of the vessel [4]. Bioresorbable scaffolds (BRSs) were conceptualized to overcome these late complications. These devices aim to provide temporary scaffolding and localized drug delivery, followed by complete resorption, ideally restoring vascular physiology and eliminating chronic foreign-body reactions. In addition, the absence of permanent metal permits non-invasive imaging and may preserve future revascularization options, such as coronary bypass grafting to previously scaffolded segments like the distal LAD [5]. Unlike permanent metallic platforms, BRSs are designed to permit late lumen enlargement, enable natural vasomotion, preserve surgical options, and facilitate non-invasive imaging once the scaffold has resorbed [6]. The distinction between polymeric and metallic BRSs is critical. Polymeric scaffolds, such as Absorb, NeoVas, and MeRes-100, utilize poly-L-lactic acid (PLLA) backbones and are designed for gradual degradation [7]. However, early-generation devices were limited by thick struts and unpredictable degradation kinetics. More recently, metallic magnesium-based scaffolds, such as Magmaris, have shown improved radial strength, faster resorption, and greater deliverability, addressing many of the mechanical limitations seen in polymeric devices [8]. Despite their conceptual appeal, BRSs have yielded mixed results in clinical trials. Outcomes have varied depending on patient selection, lesion characteristics, and operator technique. A recent review has critically appraised the limitations of early-generation devices, highlighting key failure mechanisms and underscoring the need for further innovation [9]. The concept of “transient scaffolding”—providing sufficient acute support and drug delivery, followed by scaffold resorption—remains central to ongoing innovation. A recent comprehensive review of scaffold technology highlighted the evolution of bioresorbable platforms and underscored the need for improved patient stratification, implantation technique, and device engineering [10]. In this review, we focus specifically on drug-eluting bioresorbable scaffolds (DE-BRSs), as these constitute the vast majority of clinically studied platforms to date. Building on prior insights, we analyze updated clinical outcomes and meta-analytic evidence and evaluate current challenges and future directions for next-generation platforms.

## 2. Stent Construction

The ideal BRS should provide sufficient short- to mid-term framing support to prevent restenosis and then be fully absorbed to avoid persistence of foreign materials. Therefore, it needs to offer temporary but sufficient radial support with the thinnest possible struts. The design must ensure easy delivery and manipulation, flexibility for different anatomical conditions, and structural integrity during dissolution. The optimal time frame for full resorption is still undetermined and likely device-specific, typically ranging from 12 to 36 months for polymeric platforms and faster for certain metallic systems. To meet these requirements, a wide range of materials and designs are being explored [9,10]. The two primary types of BRSs are polymer-based and metallic (Table 1).

Polymeric scaffolds. The Absorb bioresorbable vascular scaffold (BVS) consists of a poly-L-lactide (PLLA) backbone and a poly-DL-lactide (PDLLA) coating that elutes everolimus. PLLA/PDLLA degrade to lactic acid oligomers and ultimately into carbon dioxide and water via the Krebs cycle [11]. The strut thickness is ~156 μm, significantly thicker than contemporary metallic DESs, to provide initial strength. While necessary for acute radial strength, thick struts may contribute to malapposition, flow disturbance, delayed endothelialization, and thrombosis, particularly in small vessels or suboptimal expansion. These scaffolds confer radial support for approximately six months before substantial loss of mechanical integrity, with continued mass loss and resorption over two to three years. Newer polymeric devices (e.g., MeRes-100) use PLLA backbones with sirolimus elution and thinner struts (~100 μm), targeting faster endothelialization and improved acute performance with complete dissolution expected within two to three years [12].

Metallic scaffolds. Magnesium-based bioresorbable stents utilize a different technology that allows for better deliverability, more favorable elastic modulus, and a faster resorption process; they are often considered second-generation BRSs [13]. Magnesium alloys provide higher tensile strength than pure magnesium, enabling thinner struts while maintaining support. Magnesium ions may exhibit vasodilatory and anti-inflammatory properties, and the final degradation products are inorganic salts [14]. Magmaris (Biotronik, Berlin, Germany) is a balloon-expandable sirolimus-eluting magnesium scaffold with optimized alloy composition and surface treatment to modulate corrosion and drug release. While early experiences are encouraging, optimal patient/lesion selection, lesion preparation, and sizing remain crucial [15].

Other emerging materials. Beyond PDLLA/PLLA and magnesium, platforms under evaluation include tyrosine-derived polycarbonates which offer enhanced tunability of degradation and mechanical properties, poly-glycolic acid (PGA) with faster degradation, often blended for balanced kinetics, polycaprolactone (PCL) with slower degradation and enhanced flexibility, zinc-based alloys with intermediate corrosion rates and favorable biocompatibility, and iron-based scaffolds which allow for slow, predictable corrosion with potential radiopacity modifications [16,17,18]. Hybrid polymer–metal composite designs and bioresorbable shape-memory alloys are being explored to reconcile acute strength with controlled resorption and improved healing [19].

Surface engineering and drug delivery. Endothelialization-promoting nanostructures, heparin- or nitric-oxide-releasing layers, and bioactive peptide coatings seek to reduce thrombogenicity and inflammation [20]. Smart or environmentally responsive drug-delivery systems (e.g., pH- or inflammation-triggered release) aim to tailor antiproliferative elution to vascular healing dynamics, minimizing late catch-up [21].

## 3. BRS Complications

Bioresorbable scaffolds (BRSs) were designed to provide temporary vessel scaffolding, elute antiproliferative drugs, and then dissolve, ideally leaving a fully functional artery free of permanent material. However, clinical experience has revealed a spectrum of potential complications, many of which are shared with metallic DESs but others unique to the degradation process and mechanical properties of BRSs. Understanding these complications is critical for safe and effective use.

### 3.1. Scaffold Thrombosis

Among all adverse events, scaffold thrombosis remains the most feared and clinically significant. Both early and very-late thrombosis (>1 year) have been documented, particularly with first-generation polymeric devices such as Absorb. Multiple mechanisms contribute: thick struts (~150 μm) disrupt laminar flow and create zones of low shear stress, facilitating platelet aggregation and delayed endothelial coverage. Incomplete lesion preparation, underexpansion, and malapposition further expose uncovered struts to the bloodstream. Mechanical discontinuity during degradation, including strut fracture or recoil, can expose thrombogenic surfaces and serve as a nidus for thrombus formation [22,23].

Inflammatory response to degradation by-products, particularly lactic acid oligomers, may transiently lower local pH and promote fibrin deposition. Histopathologic analyses of explanted Absorb scaffolds have shown macrophage infiltration and fibrin persistence around degrading struts up to two years after implantation [22,24]. These biological phenomena, combined with suboptimal procedural technique, likely explain the higher early thrombosis rates seen in trials such as ABSORB III and AIDA.

Preventive strategies have focused on technical precision. The so-called “PSP” technique (Prepare-Size-Post-dilate) is now considered mandatory: aggressive pre-dilation to optimize lesion geometry, accurate sizing (scaffold-to-vessel ratio 1.0–1.1), and high-pressure noncompliant balloon post-dilation (>18 atm) to ensure full expansion and strut apposition. Intravascular imaging (OCT or IVUS) provides immediate feedback and should be routinely employed to detect malapposition, edge dissections, or geographic miss. When these recommendations are followed, reported thrombosis rates in newer-generation scaffolds have fallen dramatically, often below 0.5% at one year [13,25].

### 3.2. In-Scaffold Restenosis and Neoatherosclerosis

Although drug elution effectively suppresses neointimal hyperplasia, in-scaffold restenosis (ISR) remains a potential late complication, typically arising from mechanical factors. Incomplete scaffold expansion or deformation can lead to uneven drug distribution and early neointimal proliferation [26]. Moreover, as polymeric scaffolds gradually lose radial strength, vessel recoil and late lumen loss may occur, especially in small or heavily calcified vessels. Long-term imaging studies using OCT and near-infrared spectroscopy have identified neoatherosclerosis developing within the scaffolded segment before complete resorption. This process involves lipid-laden macrophage infiltration, necrotic core formation, and sometimes thin-cap fibroatheroma characteristics, findings strikingly similar to de novo atherosclerosis. It suggests that even transient implants can alter local vessel biology for several years [27]. Fortunately, next-generation scaffolds with thinner struts and improved polymer purity demonstrate lower rates of such phenomena, likely due to faster endothelial recovery and reduced chronic inflammation [28].

### 3.3. Inflammation and Delayed Vascular Healing

Biodegradation of polymeric scaffolds follows hydrolysis into lactic acid monomers, which are metabolized via the Krebs cycle. However, transient acidic microenvironments during degradation can induce local inflammation, endothelial dysfunction, and delayed re-endothelialization. In histologic analyses, persistent macrophage infiltration and microcalcification were noted in regions of late malapposition or incomplete degradation [29].

In contrast, metallic BRSs, such as magnesium-based Magmaris, show a different biological response. Magnesium ions possess intrinsic vasodilatory and anti-inflammatory properties; nevertheless, hydrogen gas release during early corrosion can produce small intramural gas pockets, occasionally visible on OCT. These are typically reabsorbed without clinical consequence, but their transient presence underscores the importance of controlled corrosion kinetics [30].

Ongoing research seeks to attenuate these inflammatory sequelae by employing composite materials, such as polycarbonate or tyrosine-derived polymers, which degrade more predictably and maintain near-neutral local pH. Surface functionalization with nitric oxide donors or endothelial cell adhesive peptides is also being explored to promote rapid endothelial recovery and minimize thrombo-inflammatory risk [31].

### 3.4. Device Malapposition and Mechanical Complications

Because most early BRSs were bulkier than metallic DESs, device malapposition emerged as a common technical challenge. First-generation BRSs also suffered from poor deliverability compared to contemporary DESs, due to bulkier profiles and limited flexibility, particularly in tortuous or calcified vessels. Inadequate expansion, edge dissections, or incomplete lesion coverage increase the risk of early restenosis or thrombosis [32,33]. Thick struts with lower hoop strength are more prone to deformation, especially in calcified, tortuous, or ostial lesions. Careful lesion preparation, including plaque modification with noncompliant balloons, cutting or scoring balloons, and, when appropriate, rotational or intravascular lithotripsy, facilitates proper scaffold expansion. After deployment, post-dilation with a noncompliant balloon matching the reference vessel diameter is essential. Underexpansion, even by 10–15%, significantly increases local flow disturbance and platelet activation [34].

Rare mechanical issues include scaffold fracture, loss, or embolization, occurring in less than 1% of cases. Embolized scaffolds may migrate distally or systemically, occasionally causing myocardial infarction or ischemic stroke. Retrieval can be attempted using snares or small balloons, while bailout metallic stenting may be necessary if repositioning fails [35,36].

### 3.5. Procedural and Patient-Related Factors

Patient-specific characteristics also influence complication risk. Small vessel diameter (<2.5 mm), diffuse disease, heavy calcification, or overlapping scaffolds are strong predictors of adverse events [37]. Likewise, premature cessation of dual antiplatelet therapy (DAPT) has been identified as a significant contributor to very-late scaffold thrombosis, particularly in real-world cohorts [38]. Clinical trials and registries such as BIOSOLVE-IV and NeoVas emphasize that when implantation follows optimized procedural guidance and DAPT is maintained for 12 months, outcomes approach those of contemporary metallic DESs [39].

## 4. Trials Comparing BVS with DESs

Up to date there are several trials comparing BRS with DES efficacy, with mixed results (Table 2). First-generation polymeric BRSs: the ABSORB program. The second-generation Absorb everolimus-eluting BVS was evaluated in ABSORB II, Cohorts B1/B2, and ABSORB EXTEND, showing favorable initial angiographic and clinical outcomes in selected lesions [40,41]. In the pivotal ABSORB III randomized trial (n = 2008), the BVS was non-inferior to cobalt-chromium everolimus-eluting stents (CoCr-EES) for one-year target lesion failure (TLF: cardiac death, target-vessel MI, or ischemia-driven TLR) [42]. However, at three years, adverse events were higher with BVS, particularly target-vessel MI and device thrombosis, highlighting vulnerability before complete resorption [43]. At five years, by which time the scaffold was essentially resorbed, the excess risk plateaued; nevertheless, the overall cumulative event rates remained higher with BVS, and commercial distribution of Absorb was discontinued [44].

ABSORB Japan. In a 2:1 randomized trial (n = 400), 12-month TLF and 13-month late lumen loss were comparable between Absorb and CoCr-EES, with low absolute thrombosis rates in both arms under rigorous procedural protocols. These findings underscored the importance of lesion selection and meticulous implantation [45].

All-comers real-world evidence: AIDA. The AIDA trial (n = 1845) randomized routine PCI patients to Absorb vs. CoCr-EES. At two years there was no significant difference in target-vessel failure (11.7% vs. 10.7%), but definite/probable device thrombosis was higher with BVS (3.5% vs. 0.9%; HR ≈ 3.9). Safety concerns prompted early reporting and contributed to withdrawal of the device. These results were pivotal in redefining safety standards and procedural requirements for BRSs [46].

Second-generation metallic BRSs: magnesium platforms. In PRAGUE-22 (ACS cohort), Magmaris was associated with greater 12-month late lumen loss versus Xience, warranting optimization of patient/lesion selection and technique [47]. In contrast, the large real-world BIOSOLVE-IV registry (n = 1075) reported encouraging 12-month outcomes with Magmaris: device success 97.3%, procedural success 98.9%, Kaplan–Meier TLF 4.3%, and definite/probable scaffold thrombosis ~0.5%, with several cases after early discontinuation of antiplatelet/anticoagulation therapy. These data support the safety and feasibility of magnesium BRSs in a lower-risk population when modern implantation standards are followed [48].

New-generation polymeric BRSs: NeoVas. In a randomized trial of 560 patients with single de novo lesions, NeoVas sirolimus-eluting BRSs demonstrated non-inferiority to CoCr-EES for one-year angiographic in-segment late loss and comparable clinical outcomes [49]. Five-year follow-up reported similar TLF between groups (9.5% vs. 7.2%; HR 1.33, *p* = 0.33) and low device thrombosis rates, with OCT showing ~72% scaffold resorption by three years and stable fractional flow reserve over time. These findings suggest meaningful progress in polymeric BRS design and healing [50].

Ultrathin BP-SES vs. contemporary DP-EES/DP-DES: implications for BRSs. Although not fully bioresorbable devices, ultrathin-strut sirolimus-eluting stents with bioabsorbable polymers (BP-SES) provide a relevant benchmark for healing and flow dynamics. A large meta-analysis of randomized trials with ≥3-year follow-up (~16,000 patients) showed comparable long-term safety and efficacy between ultrathin BP-SES and thin DP-EES across most populations, with a signal of reduced TLF in STEMI favoring BP-SES and no increased thrombotic risk [51]. A second meta-analysis similarly reported that ultrathin-strut DES were associated with a 15% reduction in long-term TLF compared with conventional second-generation thin-strut DES, while no significant differences were observed in long-term myocardial infarction (MI), stent thrombosis (ST), cardiac death, or all-cause mortality [52].

BIOSTEMI: a landmark STEMI trial of ultrathin BP-SES vs. DP-EES. In BIOSTEMI (n = 1300), patients with STEMI undergoing primary PCI were randomized to BP-SES or DP-EES. At two years, BP-SES reduced TLF (5.1% vs. 8.1%; Bayesian rate ratio 0.58) driven by lower ischemia-driven TLR, with no differences in cardiac death, target-vessel reinfarction, or definite stent thrombosis. A subgroup analysis examining complex vs. non-complex primary PCI suggested consistent benefits of BP-SES without safety trade-offs [53]. While these are not BRSs, such data inform the design goals for future BRSs: ultrathin struts, biocompatible/biodegradable polymers, and implantation standards that minimize flow disturbance and promote rapid healing [54].

Ongoing trials. The IRONMAN-II trial is evaluating imaging and physiological performance of iron-based BRSs versus current metallic DESs, including OCT-derived luminal indices and two-year late lumen loss. Results will clarify whether slow, predictable degradation with robust radial support can close the gap with best-in-class DESs [55].

## 5. Comparative Clinical Outcomes and Meta-Analytic Insights

Over the last decade, a growing body of clinical trials and meta-analyses has sought to define the relative safety and efficacy of bioresorbable scaffolds (BRSs) compared with contemporary drug-eluting stents (DESs). While early experiences with the first-generation poly-L-lactic acid (PLLA) devices raised concerns regarding thrombosis and late adverse events, the gradual evolution of scaffold design, strut thickness, and polymer behavior has led to markedly improved outcomes in the second generation of devices.

### 5.1. Evidence from Randomized Clinical Trials

Across the pivotal ABSORB program, including ABSORB II, III, and Japan, BRSs demonstrated short-term efficacy comparable to durable polymer DESs but displayed higher rates of device thrombosis during mid-term follow-up [56]. In ABSORB III, which enrolled more than 2000 patients, target-lesion failure (TLF) at 1 year was non-inferior to everolimus-eluting metallic stents, but at 3 years a higher incidence of target-vessel myocardial infarction (TV-MI) and very-late thrombosis was recorded. These findings prompted the recommendation of strict implantation techniques and ultimately led to discontinuation of the Absorb platform. Nevertheless, at the 5-year follow-up, event curves between the two devices converged, coinciding with complete scaffold bioresorption [57].

Subsequent polymeric platforms, such as NeoVas, provided more encouraging long-term outcomes. In its 5-year randomized trial, NeoVas BRSs demonstrated a TLF of 9.5% compared with 7.2% for cobalt–chromium everolimus-eluting stents (CoCr-EES), without a statistically significant difference (*p* = 0.33). Device thrombosis was low and comparable between the two groups, while OCT imaging confirmed approximately 70% resorption at three years [58].

Metal-based BRSs, particularly magnesium scaffolds, have also shown favorable short- and mid-term outcomes. The Magmaris (Biotronik) device, evaluated in the BIOSOLVE-IV registry and earlier BIOSOLVE II–III studies, achieved 12-month TLF rates around 4% and very low definite thrombosis (<0.5%), with most adverse events following premature DAPT discontinuation [59].

In acute coronary syndrome (ACS) settings, evidence from the BIOSTEMI randomized trial provided strong support for next-generation ultrathin-strut bioabsorbable-polymer sirolimus-eluting stents (BP-SES) as surrogates for full BRS designs. Among 1300 STEMI patients, BP-SES significantly reduced TLF at two years compared with durable polymer everolimus-eluting stents (5.1% vs. 8.1%) without increased thrombosis or mortality [60].

These data demonstrate that thinner struts, biodegradable polymers, and precise procedural technique can neutralize the historical safety disadvantage of early scaffolds, marking an important milestone toward the realization of a fully resorbable yet safe device.

### 5.2. Meta-Analyses and Long-Term Comparative Insights

Recent meta-analyses encompassing both polymeric and metallic platforms have provided a more global perspective. A pooled analysis of over 16,000 patients from 10 randomized controlled trials comparing BP-SES and DP-DESs revealed no statistically significant differences in long-term TLF, cardiac death, or TV-MI, though STEMI subgroups showed a modest trend toward reduced TLF with BP-SES [61].

Similarly, a meta-analysis focused exclusively on ACS patients (7 randomized studies, 7522 participants) demonstrated comparable long-term safety of ultrathin BP-SES and thin-strut DP-DESs during a mean follow-up of 41 months, confirming equivalent performance in demanding settings [62].

Network meta-analyses integrating data from both BRS and DES generations consistently rank ultrathin BP-SES and second-generation magnesium scaffolds among the top performers for combined efficacy and safety endpoints [63]. Collectively, these findings reinforce that scaffold success depends on strut architecture, degradation kinetics, and procedural accuracy rather than material alone.

### 5.3. Lessons and Implications

Event convergence after bioresorption, the evolution toward hybrid scaffold designs, and consistent findings from imaging-guided implantation underline the maturation of this technology. While procedural precision remains critical, many of the early limitations have been addressed in next-generation BRSs, bringing them closer to clinical equivalence with top-tier DESs [64].

## 6. Current Challenges and Lessons Learned

Experience from early-generation BRSs, especially the ABSORB and AIDA programs, clarified critical determinants of outcomes and informed current best practices (Table 3):

Strut thickness and flow. Thicker struts (>140–150 μm) increase flow separation and shear gradients, promoting platelet activation and delayed healing, particularly in small vessels. Next-generation BRSs target thinner struts without compromising acute strength. However, radial hoop strength is proportional to the square of strut thickness, so excessive thinning may reduce mechanical stability unless novel materials are used [65].

Lesion preparation and expansion. Underexpansion/malapposition were major contributors to events. Rigorous lesion preparation (predilation, plaque modification if needed), accurate sizing preferably with imaging guidance, and high-pressure post-dilation (PSP) are mandatory. OCT/IVUS guidance reduces geographic miss and detects edge disease, dissections, and tissue prolapse. OCT and IVUS have also been instrumental in identifying predictors of late scaffold failure and guiding vessel remodeling evaluation [66,67]. Emerging evidence also supports the use of coronary CT angiography (CCTA) for non-invasive lesion assessment and pre-procedural planning in patients considered for BRS implantation [68].

Material and degradation kinetics. Controlled, homogeneous degradation with minimal local acidity (for polyesters) and predictable loss of radial strength is essential. Magnesium alloys offer faster mass loss but require tuned corrosion control; iron-based systems provide slower, steady degradation, potentially preserving support longer [67,69].

Drug-elution strategies. Anti-proliferative agents with tailored kinetics (load, matrix, diffusion) may reduce late catch-up. Smart release responsive to inflammation or pH is under evaluation and may better synchronize with healing phases [70].

Operator training and patient selection. Outcomes improve with experience and adherence to implantation protocols. Early BRSs were used in complex anatomy (small vessels, heavy calcification) with suboptimal technique in routine practice; current guidance stresses careful selection, including vessel size (≥2.75–3.0 mm), plaque morphology, extent of calcification, and overall lesion complexity, until devices mature [71,72].

Antithrombotic therapy. Premature discontinuation of DAPT was a trigger in several early scaffold thrombosis cases. Individualized DAPT duration balancing bleeding/ischemic risk remains important, and ongoing studies will refine recommendations for specific platforms [73].

Collectively, these lessons explain the initial shortfalls and the steady improvement seen with newer platforms and optimized practice. They also emphasize that BRSs are a system comprising device, technique, patient, and pharmacotherapy—not a device alone. Importantly, differences in implantation protocols between study arms, including more intensive lesion preparation and imaging guidance for BRSs, may influence comparative outcomes and should be considered in interpreting trial data.

## 7. Next-Generation Scaffolds and Future Directions

The continuous evolution of coronary stent technology has given rise to a new generation of bioresorbable scaffolds (ng-BRSs), aiming to address mechanical, biological, and procedural limitations of earlier designs. Cost also remains a major barrier to widespread adoption of BRSs. Without clear clinical superiority over contemporary DESs, the higher price of bioresorbable scaffolds has limited their integration into routine clinical practice, especially in cost-sensitive healthcare systems [74]. The limited commercial uptake has also constrained operator experience, which in turn affects optimal implantation and broader outcome evaluation.

Innovative biomaterials. Tyrosine-derived polycarbonate scaffolds and iron-based BRSs are gaining attention. Tyrosine-derived materials provide enhanced flexibility and tunable biodegradation; iron-based scaffolds, currently evaluated in the IRONMAN-II trial, offer slow, predictable degradation with robust radial support and potential for improved radiopacity [75].

Adaptive/smart drug delivery. Systems with release timing coupled to local inflammatory markers or pH aim to provide context-sensitive anti-proliferative therapy, potentially reducing late events while preserving healing [76].

Imaging-guided implantation and AI-assisted PCI. Routine use of OCT/IVUS for lesion assessment, sizing, and post-deployment optimization reduces malapposition and thrombosis [77]. AI-assisted platforms that integrate angiography, intravascular imaging, and computational modeling may standardize sizing and expansion targets, reduce operator variability, and enhance outcomes [78].

Surface nanostructuring and hybrid designs. Nanotopographies that mimic endothelial basement membrane, nitric-oxide releasing surfaces, and polymer-metal composites are under active investigation to accelerate endothelial coverage and reduce thrombogenicity [79].

The evolving role of drug-eluting balloons (DEBs) in PCI raises additional questions about the complementary or competitive positioning of BRSs, especially in bifurcation or small-vessel disease where permanent implants are less desirable [80].

As bioresorbable technologies mature, a realistic expectation is incremental improvement rather than abrupt replacement of modern DESs. Near-term milestones include demonstration of non-inferiority in carefully selected lesions under imaging-guided protocols, with longer-term goals of equal or superior outcomes driven by better restoration of vasomotion and reduced late events [81,82].

## 8. Conclusions

**Figure 1:**
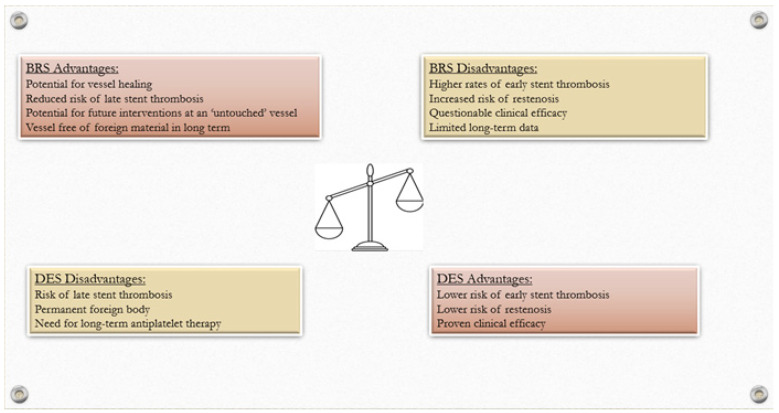
Main advantages and disadvantages of bioresorbable scaffolds (BRSs) compared to drug-eluting stents (DESs).

While coronary stenting remains the standard treatment for patients undergoing angioplasty, the emergence of BRS technology presents an innovative alternative that holds promise for the future of CAD management. However, the technology is still evolving, and important challenges remain (Figure 1). Early-generation polymeric BRSs were limited by thick struts, suboptimal implantation techniques, and unfavorable risk profiles in unselected populations, as highlighted by pivotal randomized trials and real-world evidence. Newer polymeric and metallic designs, together with rigorous lesion preparation, accurate imaging-guided sizing, and standardized post-dilation, are narrowing the gap with best-in-class DESs.

Recent data, from magnesium-based registries, newer PLLA platforms (NeoVas), and ultrathin bioabsorbable-polymer DES benchmarks (e.g., BIOSTEMI), suggest that the foundational principles of transient scaffolding are sound when executed with appropriate materials, optimized pharmacology, and precise technique. Ongoing trials (e.g., iron-based platforms) and advances in materials science, drug delivery, surface engineering, and AI-assisted PCI will further define the role of BRSs. Until robust long-term equivalence or superiority is consistently demonstrated across broader lesion subsets, a cautious, evidence-based, and imaging-guided approach to BRS implantation is warranted.

## Figures and Tables

**Table 1:** Representative drug-eluting bioresorbable scaffolds (DES-BRSs) and their key characteristics.

Platform	Backbone Material	Drug	Nominal Strut Thickness	Key Features/Considerations
Absorb BVS	PLLA + PDLLA coating	Everolimus	~156 μm	First-generation polymeric BRS; thick struts; learning-curve dependent
MeRes-100	PLLA	Sirolimus	~100 μm	Thinner struts vs. Absorb; imaging-guided optimization recommended
Magmaris	Mg alloy	Sirolimus	~150 μm (design-dependent)	Second-generation metallic BRS; favorable handling; registry data supportive
NeoVas	PLLA (modified)	Sirolimus	~100–120 μm	New-generation PLLA BRS; 5-year outcomes vs. CoCr-EES comparable in noncomplex lesions
Iron-based BRS	Fe alloy	Sirolimus (some)	Under study	Robust radial support; ongoing RCTs (e.g., IRONMAN-II)

**Table 2:** Key trials and meta-analyses comparing BRS/BP-SES with DES.

Study	Design/n	Population	Comparator(s)	Main Findings
ABSORB III	RCT, n = 2008	CAD	Absorb vs. CoCr-EES	1-y non-inferiority; ↑ events at 3 y (TV-MI, thrombosis); plateau after ~3 y
ABSORB Japan	RCT, n = 400	De novo lesions	Absorb vs. CoCr-EES	Similar TLF/LLL; low thrombosis with optimized technique
AIDA	All-comers RCT, n = 1845	Routine PCI	Absorb vs. CoCr-EES	No TVF difference; ↑ definite/probable thrombosis with BRS
PRAGUE-22	RCT, ACS	ACS	Magmaris vs. Xience	↑ late lumen loss with Mg-BRS
BIOSOLVE-IV	Registry, n = 1075	Real-world	Magmaris	TLF 4.3%; thrombosis ~0.5%; high device/procedural success
NeoVas RCT	RCT, n = 560	Single de novo	NeoVas vs. CoCr-EES	Non-inferior 1-y LLL; 5-y TLF comparable; ~72% resorption at 3 y
BIOSTEMI	RCT, n = 1300	STEMI (pPCI)	BP-SES vs. DP-EES	↓ TLF with BP-SES (driven by TLR); similar death/MI/thrombosis
Meta-analysis 1	RCTs, n ≈ 16,000	PCI (mixed)	Ultrathin BP-SES vs. DP-EES	Comparable long-term outcomes; STEMI signal favoring BP-SES
Meta-analysis 2	RCTs, n = 7522	ACS only	Ultrathin BP-SES vs. thin DP-DES	Comparable TLF, CD, TV-MI, CD-TLR

↑: increased risk, ↓: decreased risk.

**Table 3:** Current challenges and strategies to overcome them.

Challenge	Mechanism/Manifestation	Mitigation Strategies
Thick struts and flow disturbance	Delayed endothelialization, thrombosis	Thinner struts; optimized alloy/polymer; smoother profiles
Underexpansion/malapposition	Edge dissections, tissue prolapse	PSP technique; routine OCT/IVUS for sizing and optimization
Unfavorable degradation kinetics	Local acidity/inflammation (polymers)	Copolymers/blends; buffering layers; corrosion-controlled metals
Insufficient radial strength in complex lesions	Recoil/fracture risk	Patient/lesion selection; hybrid designs; staged PCI
Late neoatherosclerosis	Lipid-rich neointima	Pro-healing coatings; sustained/targeted drug elution
DAPT management	Early cessation → events	Protocolized DAPT; risk-tailored duration; patient education

## Data Availability

No new data were created or analyzed in this study. Data sharing is not applicable to this article.
